# Implementation of national action plans on noncommunicable diseases, Bhutan, Cambodia, Indonesia, Philippines, Sri Lanka, Thailand and Viet Nam

**DOI:** 10.2471/BLT.18.220483

**Published:** 2018-12-19

**Authors:** Titiporn Tuangratananon, Sangay Wangmo, Nimali Widanapathirana, Suladda Pongutta, Shaheda Viriyathorn, Walaiporn Patcharanarumol, Kouland Thin, Somil Nagpal, Christian Edward L Nuevo, Retna Siwi Padmawati, Maria Elizabeth Puyat-Murga, Laksono Trisnantoro, Kinzang Wangmo, Nalinda Wellappuli, Phuong Hoang Thi, Tuan Khuong Anh, Thinley Zangmo, Viroj Tangcharoensathien

**Affiliations:** a International Health Policy Program, Ministry of Public Health, Tivanond Road, Muang District, Nonthaburi 11000, Thailand.; b Nutrition and Indigenous Medicine, Ministry of Health, Colombo, Sri Lanka.; c Swiss Agency for Development and Cooperation, Phnom Penh, Cambodia.; d Global Practice on Health, Nutrition and Population, World Bank, Phnom Penh, Cambodia.; e Department of Health, Manila, Philippines.; f Faculty of Medicine, Public Health and Nursing, Universitas Gadjah Mada, Yogyakarta, Indonesia.; g Health Sciences Programme, Ateneo de Manila University, Manila, Philippines.; h School of Medicine, Gadjah Mada University, Yogyakarta, Indonesia.; i Policy and Planning Division, Ministry of Health, Thimphu, Bhutan.; j Management Development and Planning Unit, Ministry of Health, Colombo, Sri Lanka.; k Health Strategy and Policy Institute, Ministry of Health, Hanoi, Vietnam.; l Health Promotion Division, Ministry of Health, Thimpu, Bhutan.

## Abstract

By 2016, Member States of the World Health Organization (WHO) had developed and implemented national action plans on noncommunicable diseases in line with the *Global action plan for the prevention and control of noncommunicable diseases (2013–2020).* In 2018, we assessed the implementation status of the recommended best-buy noncommunicable diseases interventions in seven Asian countries: Bhutan, Cambodia, Indonesia, Philippines, Sri Lanka, Thailand and Viet Nam. We gathered data from a range of published reports and directly from health ministries. We included interventions that addressed the use of tobacco and alcohol, inadequate physical activity and high salt intake, as well as health-systems responses, and we identified gaps and proposed solutions. In 2018, progress was uneven across countries. Implementation gaps were largely due to inadequate funding; limited institutional capacity (despite designated noncommunicable diseases units); inadequate action across different sectors within and outside the health system; and a lack of standardized monitoring and evaluation mechanisms to inform policies. To address implementation gaps, governments need to invest more in effective interventions such as the WHO-recommended best-buy interventions, improve action across different sectors, and enhance capacity in monitoring and evaluation and in research. Learning from the Framework Convention on Tobacco Control, the WHO and international partners should develop a standardized, comprehensive monitoring tool on alcohol, salt and unhealthy food consumption, physical activity and health-systems response.

## Résumé

En 2016, les États membres de l'Organisation mondiale de la Santé (OMS) avaient élaboré et mis en œuvre des plans d'action nationaux sur les maladies non transmissibles conformément au Plan d'action mondial pour la lutte contre les maladies non transmissibles (2013–2020). En 2018, nous avons évalué l'état de l'application des interventions les plus avantageuses recommandées en matière de maladies non transmissibles dans sept pays asiatiques: le Bhoutan, le Cambodge, l'Indonésie, les Philippines, le Sri Lanka, la Thaïlande et le Viet Nam. Nous avons recueilli des données à partir de toute une série de rapports publiés et directement auprès des ministères de la Santé. Nous avons inclus les interventions qui concernaient la consommation de tabac et d'alcool, une activité physique inadéquate et une consommation de sel élevée, ainsi que les réponses des systèmes de santé, et nous avons identifié les lacunes et proposé des solutions. En 2018, les progrès étaient variables selon les pays. Les lacunes étaient largement dues à un financement inadéquat; des capacités institutionnelles limitées (malgré des unités dédiées aux maladies non transmissibles); une action inadéquate dans les différents secteurs au sein et en dehors du système de santé; et l'absence de mécanismes de suivi et d'évaluation standardisés pour orienter les politiques. Afin de combler ces lacunes, les gouvernements doivent investir davantage dans des interventions efficaces telles que les interventions les plus avantageuses recommandées par l'OMS, améliorer l'action dans les différents secteurs, et renforcer les capacités en matière de suivi et d'évaluation, mais aussi de recherche. En s'inspirant de la Convention-cadre pour la lutte antitabac, l'OMS et ses partenaires internationaux devraient élaborer un outil de suivi complet et standardisé sur la consommation d'alcool, de sel et d'aliments malsains, l'activité physique et la réponse des systèmes de santé.

## Resumen

Para 2016, los Estados miembros de la Organización Mundial de la Salud (OMS) habían elaborado y aplicado planes de acción nacionales sobre las enfermedades no contagiosas de acuerdo con el Plan de acción mundial para la prevención y el control de las enfermedades no transmisibles (2013-2020). En 2018, se evaluó el estado de implementación de las intervenciones recomendadas en siete países asiáticos en materia de enfermedades no contagiosas: Bhután, Camboya, Filipinas, Indonesia, Sri Lanka, Tailandia y Vietnam. Se recopilaron datos de una serie de informes publicados y directamente de los ministerios de salud. Se incluyeron intervenciones que abordaron el uso del tabaco y el alcohol, la actividad física inadecuada y la ingesta elevada de sal, así como las respuestas de los sistemas de salud, se identificaron las deficiencias y se propusieron soluciones. En 2018, el progreso fue desigual entre los países. Las deficiencias en la aplicación se debieron en gran medida a la falta de financiación, a la limitada capacidad institucional (a pesar de las dependencias designadas para las enfermedades no contagiosas), a la inadecuación de las medidas adoptadas en los diferentes sectores dentro y fuera del sistema de salud y a la falta de mecanismos normalizados de supervisión y evaluación que sirvieran de base a las políticas. Para subsanar las deficiencias en materia de aplicación, los gobiernos deben invertir más en intervenciones eficaces, como las recomendadas por la OMS, mejorar las medidas adoptadas en los distintos sectores y aumentar la capacidad de seguimiento y evaluación y de investigación. A partir de las enseñanzas del Convenio Marco para el Control del Tabaco, la OMS y los asociados internacionales deberían elaborar un instrumento de seguimiento normalizado y completo para el consumo de alcohol, sal y alimentos no saludables, la actividad física y la respuesta de los sistemas de salud.

## ملخص

قامت الدول الأعضاء في منظمة الصحة العالمية (WHO) في عام 2016 بتطوير وتنفيذ خطط عمل وطنية بشأن الأمراض غير المعدية بما يتماشى مع خطة العمل العالمية للوقاية من الأمراض غير المعدية ومكافحتها (2013-2020). في عام 2018، قمنا بتقييم حالة تنفيذ أفضل التدخلات الموصى في الأمراض غير المعدية في سبعة بلدان آسيوية: إندونيسيا والفلبين وبوتان وتايلند وسري لانكا وفييت نام وكمبوديا. قمنا بجمع بيانات من مجموعة من التقارير المنشورة، كما جمعنا البيانات مباشرة من وزارات الصحة. وقمنا بتضمين التدخلات التي تناولت استخدام التبغ والكحول، والنشاط البدني غير الكافي والاستهلاك المرتفع من الملح، وكذلك استجابات الأنظمة الصحية، وحددنا الفجوات والحلول المقترحة. وفي عام 2018، كان التقدم متفاوتا بين البلدان. وكانت الفجوات في مستوى التنفيذ ترجع إلى حد كبير إلى عدم كفاية التمويل؛ والقدرات المؤسسية المحدودة (على الرغم من الوحدات المخصصة للأمراض غير السارية)؛ وعدم كفاية العمل عبر القطاعات المختلفة داخل وخارج النظام الصحي؛ وعدم وجود آليات موحدة للرصد والتقييم لتوجيه السياسات. ولمعالجة الفجوات على مستوى التنفيذ، تحتاج الحكومات إلى أن تستثمر أكثر في التدخلات الفعالة مثل أفضل التدخلات التي توصى بها منظمة الصحة العالمية، وتحسين العمل عبر مختلف القطاعات، وتعزيز القدرة على الرصد والتقييم في الأبحاث. بناء على الاستفادة المحققة من الاتفاقية الإطارية لمكافحة التبغ، فإنه يجب على كل من منظمة الصحة العالمية والشركاء الدوليين تطوير أداة رصد قياسية وشاملة لكل من الكحول والملح والاستهلاك الغذائي غير الصحي والنشاط البدني واستجابة النظم الصحية.

## 摘要

截至 2016 年，世界卫生组织 (WHO) 成员国均已根据《预防和控制非传染性疾病全球行动计划 (2013-2020)》开展并实施了非传染性疾病国家行动计划。2018 年，我们评估了亚洲七国预防和控制非传染性疾病的“最合算措施”以及其它推荐干预措施的实施情况。这七个国家分别是：不丹、菲律宾、柬埔寨、斯里兰卡、泰国、印度尼西亚、和越南。我们从一系列已发表的报告和卫生部门直接收集数据。调查涵盖了减少烟草使用、减少有害使用酒精、减少身体不足活动、减少高盐摄入等干预措施，同时还有卫生系统反应，我们由此确定实施的差距，并提出解决方案。2018 年，各国在此方面的进展并不均衡。干预措施的实施存在差距的主要原因包括资金不足；机构能力有限（尽管指派了非传染性疾病部门）；卫生系统内外不同部门的行动不足；以及缺乏制定政策的标准化监测和评估机制。为了解决实施差距，政府应更多地采取有效的干预措施，例如世界卫生组织预防和控制非传染性疾病的“最合算措施”以及其它推荐干预措施，从而改善不同部门的行动力，提高监测、评估和研究的能力。根据《烟草控制框架公约》，世卫组织及其国际合作伙伴应制定关于酒精、盐和不健康饮食、身体活动不足和卫生系统反应的标准化综合监测工具。

## Резюме

К 2016 году страны-члены Всемирной организации здравоохранения (ВОЗ) разработали и осуществили национальные планы действий в отношении неинфекционных заболеваний в соответствии с Мировым планом действий по предотвращению и контролю распространения неинфекционных заболеваний (2013–2020 гг.). В 2018 году была проведена оценка состояния рекомендуемых и наиболее популярных мер борьбы с неинфекционными заболеваниями в семи странах Азии: в Бутане, Вьетнаме, Индонезии, Камбодже, Таиланде, на Филиппинах и в Шри-Ланке. Были собраны данные ряда опубликованных отчетов, а также получены сведения непосредственно из министерств здравоохранения. Авторы включили в обзор действия в отношении употребления табака и алкоголя, борьбы с недостаточной физической активностью и высоким потреблением соли, а также оценили реакцию систем здравоохранения, выявили недостатки системы действий и предложили способы их устранения. По состоянию на 2018 год страны демонстрировали неравномерный прогресс. Основные недостатки предпринятых действий были связаны с недостаточным финансированием, ограниченными институциональными возможностями (несмотря на наличие специально созданных отделов по борьбе с неинфекционными заболеваниями), недостаточностью действий в разных секторах внутри системы здравоохранения и вне ее, а также с нехваткой стандартизированных механизмов мониторинга и оценки для информирования лиц, принимающих стратегические решения. Для ликвидации отставания правительства должны больше инвестировать в эффективные меры борьбы, которые рекомендованы ВОЗ как наиболее популярные, улучшать взаимодействие секторов и расширять возможности исследований, мониторинга и оценки. Опираясь на опыт Рамочной конвенции по борьбе против табака, ВОЗ и ее международные партнеры должны разработать стандартизированный всеобъемлющий метод мониторинга потребления алкоголя, соли и вредных продуктов питания, а также оценки физической активности и реакции системы здравоохранения.

## Introduction

Noncommunicable diseases, such as cardiovascular diseases, cancers, chronic respiratory diseases and diabetes, claim a high proportion of overall mortality, pushing many people into poverty due to catastrophic spending on medical care.^1^ Yet noncommunicable diseases are mostly preventable. The United Nations (UN) General Assembly has adopted a series of resolutions^2^ which reflect the high-level commitment to prevention and control of noncommunicable diseases. In 2013, Member States of the World Health Organization (WHO) resolved to develop and implement national action plans, in line with the policy options proposed in the *Global action plan for the prevention and control of noncommunicable diseases (2013–2020)*.^3^ Noncommunicable diseases are also embedded in sustainable development goal (SDG) target 3.4, that is, to reduce by one-third the premature mortality from noncommunicable diseases by 2030, and are linked to other SDGs, notably SDG 1 to end poverty.^4^ In 2017, the WHO Global Conference on Noncommunicable Diseases^5^ reaffirmed noncommunicable diseases as a sustainable development priority in the *Montevideo roadmap 2018–2030*.^6^

The WHO estimates an economic return of 7 United States dollars (US$) per person for every dollar spent on so-called best buys – evidence-based, highly cost–effective policy interventions which tackle noncommunicable diseases.^7^ There could also be a reduction of 8.1 million premature deaths by 2030 if these best-buy options were fully implemented, which represents 15% of the total premature deaths due to noncommunicable diseases.^7^ Despite the rising burden of these diseases in low- and middle-income countries, only an estimated 1% of health funding in these countries is dedicated to prevention and clinical management.^7^ This level of spending is unlikely to have a significant impact.

Country-level gaps in legislative, regulatory, technical and financial capacities impede the translation of global commitments into national action. Most low- and middle-income countries have weak health systems, with limited domestic and international funding for prevention and health promotion interventions. Between 2000 and 2015, only 1.3% (US$ 5.2 billion) of total global development assistance for health was contributed to noncommunicable disease programmes.^8^ The problems are compounded by a lack of coordinated action across the relevant sectors within and outside governments.^9^^–^^11^ WHO has recommended that innovative sources of domestic financing be explored.^12^ Yet in most low- and middle-income countries, inadequate government funding and high out-of-pocket payments often prevent poorer people from accessing treatment for noncommunicable diseases.^8^^,^^13^

We assessed the implementation status of best-buy interventions in seven Asian countries, which have participated in collaborative studies of noncommunicable diseases: Bhutan, Cambodia, Indonesia, Philippines, Sri Lanka, Thailand and Viet Nam. We also assessed gaps in institutional capacity and provided suggestions for improving policy implementation. All countries in this analysis are currently classified by the World Bank as lower-middle income, except Thailand, which is classified as upper-middle income.^14^ Population size ranges from under 1 million in Bhutan to more than 250 million in Indonesia. There are large variations in the prevalence of risk factors for noncommunicable disease, its associated burden and measures to tackle them across these seven countries (Table 1). 

**Table 1:** Profile of seven Asian countries included in the analysis of best-buy interventions for the prevention and control of noncommunicable diseases in July 2018

Variable	Bhutan	Cambodia	Indonesia	Philippines	Sri Lanka	Thailand	Viet Nam
Total population, millions in 2017	0.8	16	258	102	21	69	94 (2016)
**Economic and fiscal measures**^15^							
GDP per capita in 2017, current US$	3110	1384	3847	2989	4065	6594	2343
Government revenue, excluding grants in 2016, % of GDP	18.9	17.4	12.5	15.2	14.2	20.0	21.5 (2013)
**Health expenditure**^15^							
Current health expenditure per capita in 2015, current US$	91	70	112	127	118	217	117
**Physical activity indicators**^16^							
Prevalence of physical activity by adults age 18+ years in 2013, %							
Both sexes	91	NA	76	NA	76	70	76
Males	94	NA	75	NA	83	68	78
Females	88	NA	78	NA	70	72	74
Estimated deaths related to physical inactivity in 2013, %	14.0	NA	8.0	NA	6.9	5.1	4.1
**Alcohol indicators**^17^							
Total alcohol consumption per capita by alcohol drinkers older than 15 years in 2010, litres of pure alcohol	6.9	14.2	7.1	12.3	20.1	23.8	17.2
National legal minimum age for on-premise sales of alcoholic beverages, years	18	None	None	18	21	20	18
National maximum legal blood alcohol concentration, %	0.08	0.05	Zero	0.05	0.08	0.05	Zero
**Tobacco indicators**^18^							
WHO FCTC, year of signatory; year of ratification	2003; 2004	2004; 2005	Not signed or ratified	2003; 2005	2003; 2003	2003; 2004	2003; 2004
Prevalence of tobacco use among young people aged 13–15 years in 2016, %							
Both sexes	30.2	2.4	12.7	12.0	3.7	15.0	4.0
Males	39.0	2.9	23.0	17.6	6.7	21.8	6.9
Females	23.2	1.9	2.4	7.0	0.7	8.1	1.3
Prevalence of tobacco smoking among individuals older than 15 years in 2016, %							
Both sexes	7.4	21.8	NA	22.7	15.0	20.7	22.5
Males	10.8	33.6	64.9	40.3	29.4	40.5	45.3
Females	3.1	11.0	2.1	5.1	0.1	2.2	1.1
Total tobacco taxes, % of retail price	Tobacco banned	25.2	57.4	62.6	62.1	73.5	35.7

FCTC: Framework Convention on Tobacco Control; GDP: gross domestic product; NA: data unavailable; US$: United States dollar.

Although these seven countries have a similar pace of socioeconomic development, they are diverse in terms of population size, health-system structure and decentralization of governance for health (fully devolved to local governments in Indonesia and the Philippines, and partially devolved in Sri Lanka). Lessons from their experiences can be shared with other countries striving to implement their national action plans on noncommunicable diseases.

## Approach

**Fig. 1:**
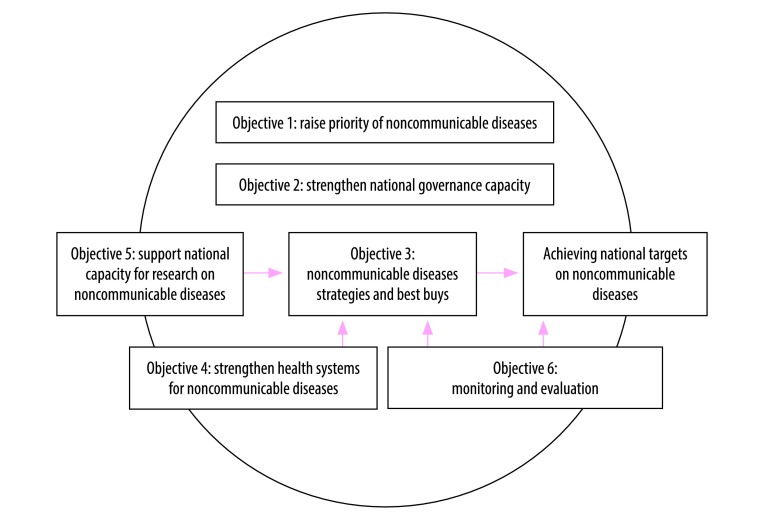
Noncommunicable diseases global action plan framework: the interlinks between six objectives in achieving national targets on noncommunicable diseases

Note: Based on the *WHO Global action plan for the prevention and control of noncommunicable diseases 2013–2020*.^3^

We based our analysis on the policy options in the six objectives in the global action plan on noncommunicable dieases.^3^ These objectives form the guiding framework for WHO Member States to develop their national action plans (Fig. 1). National research capacities (objective 5) and monitoring and evaluation (objective 6) provide evidence which supports the application of best-buy interventions (objective 3) and monitors progress towards achieving targets. Health-systems strengthening (objective 4) supports the implementation of the action plan. All four objectives (3, 4, 5 and 6) should be enhanced by good governance (objective 2) and a heightened noncommunicable diseases priority that sustains the agenda across successive governments (objective 1).

Given the six objectives act in synergy to contribute to noncommunicable diseases prevention and control, we did not attempt to address all of them, but to focus on implementation of the best buys for four major noncommunicable diseases risk factors (tobacco, alcohol, unhealthy diet and physical activity) and for health-systems response.

In the first half of 2018, we gathered information from country profiles in a range of sources from the published literature: (i) the *WHO report on the global tobacco epidemic 2017* which was compiled by the Framework Convention on Tobacco Control (FCTC) secretariat;^18^ (ii) the WHO *Global status report on alcohol and health 2018*;^19^ (iii) the *WHO Global status report on noncommunicable diseases 2010*;^20^ (iv) the *Noncommunicable diseases progress monitor 2017*;^21^ (v) national capacity survey data on physical activity, salt policy and health-systems response to developing treatment guidelines from the WHO *Global Health Observatory data repository*;^22^ and (vi) the *Noncommunicable diseases country profiles 2018* report on availability of essential medicines for noncommunicable diseases.^23^ Additional published literature was retrieved from a search of PubMed® and Scopus online databases. We used personal contacts with the health ministries in each respective country to obtain further information on the institutional capacity to address noncommunicable diseases.

## Implementation of best buys

Table 2 provides a summary of the implementation status of best-buy interventions across the seven countries.

**Table 2:** Implementation status of best-buy interventions for the prevention and control of noncommunicable diseases in seven Asian countries in July 2018

Best-buy intervention	Indicator description	Bhutan	Cambodia	Indonesia	Philippines	Sri Lanka	Thailand	Viet Nam
**Tobacco demand-reduction measures**^18^								
1. Increase excise taxes and prices on tobacco products	Total taxes as % of the price of the most sold brand of cigarettes was maximum 75% and above, minimum 51%^24^	Not applicable, as sale of tobacco banned in Bhutan	Total tax: 25.2% of retail price in 2016. Retail cigarette price affordable. No changes between 2008 and 2016	Total tax: 57.4% of retail price in 2016. Retail cigarette price affordable. Cigarettes more affordable in 2016 than 2008	Total tax: 62.6% of retail cigarette price in 2016. Cigarettes less affordable in 2016 than 2008	Total tax: 62.1% of retail cigarette price in 2016. Tobacco price affordable. No changes between 2008 and 2016	Total tax: 73.5% of retail price in 2016. Retail cigarette price affordable. No changes between 2008 and 2016	Total tax: 35.7% of retail cigarette price in 2016. Cigarettes more affordable in 2016 than in 2008
2. Eliminate exposure to second-hand tobacco smoke in all indoor workplaces, public places and public transport	Compliance score for smoke-free environments as per WHO report.^18^ High compliance: 8–10; moderate compliance: 3–7; minimal compliance: 0–2	Compliance score: 10/10 in 2016. Not yet enforced compliance in cafés, pubs, bars, government facilities and universities	Compliance score: 5/10 in 2016. Not yet enforced compliance in restaurant and government facilities	Compliance score: 1/10 in 2016. Not yet introduced smoke-free regulation in government facilities, indoor offices, restaurant, cafés, pubs and bars	Compliance score: 5/10 in 2016. Not yet introduced smoke-free regulation in indoor offices, restaurants, cafés, pubs and bars	Compliance score: 6/10 in 2016. Not yet introduced smoke-free regulation in restaurants, cafés, pubs and bars	Compliance score: 7/10 (score from 2013 MPOWER report^25^). Complete compliance with smoke-free regulation in health-care facilities, educational facilities, universities, government facilities, indoor offices, restaurants, cafés, pubs and bars and public transport	Compliance score: 5/10 in 2016. Not yet introduced smoke-free regulation in café, pubs, bars and public transport
3. Implement plain or standardized packaging and/or large graphic health warnings on all tobacco packages	Mandates plain or standardized packaging or large graphic warnings with all appropriate characteristics	Not applicable	Mandates pictorial and text health warnings on packaging of cigarettes, other smoked tobacco and smokeless tobacco, covering 55% of front and back areas. Two specific health warning approved	Mandates pictorial and text health warnings on packaging of cigarettes, other smoked tobacco and smokeless tobacco, covering 40% of front and back areas. Five specific health warnings approved	Mandates pictorial and text health warnings on packaging of cigarettes, other smoked tobacco and smokeless tobacco, covering 50% of front and back areas. Twelve specific health warnings approved	Mandates text and pictorial health warnings on packaging of cigarettes and other smoked tobacco, covering 80% of front and back areas. (Ban on smokeless tobacco.) Four specific health warnings approved	Mandates text and pictorial health warnings on packaging of cigarettes and other smoke tobacco, covering 85% of front and back areas. Ban on smokeless tobacco. Ten specific health warnings approved	Mandates text and pictorial health warnings on packaging of cigarettes, other smoked tobacco and smokeless tobacco, covering 50% of front and back areas. Six specific health warnings approved
4. Enact and enforce comprehensive bans on tobacco advertising, promotion and sponsorship	Compliance score as per WHO report.^18^ High compliance: 8–10; moderate compliance: 3–7; minimal compliance: 0–2	Compliance score on direct advertising ban: 10/10; promotions and sponsorship ban: 10/10; indirect promotions ban: 10/10	Compliance score on direct advertising ban: 8/10. No ban on indirect promotions except on publicizing corporate social responsibility activities of tobacco companies	No ban on direct tobacco advertising in TV or radio, magazines, billboards, point-of-sales or the internet. Compliance score on free distribution ban: 3/10; promotional discounts on television ban: 0/10; non-tobacco products identified with tobacco brand names ban: 1/10	Compliance score on direct advertising ban: 6/10. No ban on promotions except appearance of tobacco brands on television or films (product placement) score: 9/10; indirect promotions ban: 6/10	Compliance score on direct advertising ban: 8/10; promotions ban: 5–10/10; indirect promotions ban: 6/10	Comprehensive regulations on advertising, market promotion and sponsorship, and indirect promotions (no score reported in 2017 WHO MPOWER report^25^)	Compliance score on direct advertising ban: 10/10; promotions ban: 6–8/10; indirect promotions ban: 6/10
5. Implement effective mass-media campaigns that educate the public about the harms of smoking/tobacco use and second-hand smoke	Implemented a national anti-tobacco mass-media campaign designed to support tobacco control, of at least 3 weeks duration with all appropriate characteristics^24^	No national media campaign implemented between 2014 and 2016	National media campaign implemented on television and radio between 2014 and 2016. Content and target audience guided by research, though no post-campaign evaluation was made	Media campaign implemented between 2014 and 2016. Content and target audience guided by research, with post-campaign evaluation	Comprehensive media campaign implemented between 2014 and 2016. Content and target audience guided by research, with post-campaign evaluation	No media campaign implemented between 2014 and 2016	Comprehensive media campaign implemented between 2014 and 2016. Content and target audience guided by research, with post-campaign evaluation	Comprehensive media campaign implemented between 2014 and 2016. Content and target audience guided by research, with post-campaign evaluation
**Harmful use of alcohol reduction measures**^19^								
1. Enact and enforce restrictions on the physical availability of retailed alcohol (via reduced hours of sale)	National legal minimum age for on- and off-premise sales of alcoholic beverages^19^	18 years	No defined legal age	21 years	18 years	21 years	20 years	18 years
	Restrictions for on- and off-premise sales of alcoholic beverages by hours, days, places of sale, density of outlets, for specific events, to intoxicated persons, at petrol stations^19^	Restrictions for all categories except density	No restrictions	Restrictions only for hours and places	Restrictions only for hours, places, density and specific events	Restrictions for all categories	Restrictions for all categories except density and specific events	Restrictions only by place, density and for intoxicated persons
2. Enact and enforce bans or comprehensive restrictions on exposure to alcohol advertising (across multiple types of media)	Legally binding regulations on alcohol advertising, product placement, sponsorship, sales promotion, health warning labels on advertisements and containers	Yes, except advertising on containers	Regulations only on alcohol sponsorship	Yes, except advertising on containers	Regulations only for health warning labels on alcohol advertisements and containers	Yes, except advertising on containers	Yes, except advertising on containers	Yes, except advertising on containers
3.Increase excise taxes on alcoholic beverages	Excise tax on beer, wine and spirits	Yes, except for spirits	Yes	Yes	Yes	Yes	Yes	Yes
**Unhealthy diet reduction measures**^22^								
1. Adopt national policies to reduce population salt/sodium consumption	Adopted national salt policies	No	No	No	No	No	Yes	No
	Applies voluntary or mandatory salt cut-offs on selected foods	No	No	No	No	No	Applies voluntary salt reduction in processed food and snacks with healthier choice logo. Mandatory regulation for food labelling in guideline daily amounts	No
**Physical activity**^22^								
1. Implement communitywide public education and awareness campaign for physical activity, which includes a mass media campaign	Country has implemented, within past 5 years, at least one recent national public awareness programme on physical activity	Yes	No	Yes	Yes	Yes	Yes	No
**Health systems**^24^								
1. Member State has national management guidelines for four major noncommunicable diseases through a primary care approach	Availability of national guidelines for the management of cardiovascular diseases, diabetes, cancer and chronic respiratory diseases	Yes	Yes	Yes	Yes	Yes	Yes	Yes
2. Drug therapy for diabetes mellitus and hypertension using total risk approach), and counselling to individuals who have had a heart attack or stroke and to persons with high risk (≥ 30%, or ≥ 20%) of a fatal and non-fatal cardiovascular event in the next 10 years	Proportion of primary health-care facilities offering cardiovascular risk stratification for the management of patients at high risk for heart attack and stroke^23^	Less than 25%	Less than 25%	Less than 25%	More than 50%	More than 50%	More than 50%	Less than 25%
	Availability of selected noncommunicable diseases medicines at 50% or more of primary-health care facilities^22^	4/12 drugs	3/12 drugs	11/12 drugs	4/12 drugs	11/12 drugs	9/12 drugs	2/12 drugs

WHO: World Health Organization.

Note: Affordability of cigarettes is defined by the percentage of per capita gross domestic product required to purchase 2000 cigarettes of the most sold brand.^18^

### Tobacco control

All six countries that are State Parties to the WHO FCTC,^18^ and also Indonesia, which is not a State Party to the Convention, have implemented tobacco control interventions. There are five indicators to monitor progress as mandated by the Convention. 

First, countries are required to increase excise taxes and prices on tobacco products to achieve the total tax rate between 51% and 75% of retail price of the most sold brand of cigarettes. By 2016, no country in our analysis had achieved the target of 75%. Thailand had the highest tax rate of 73.5%, while Cambodia had the lowest rate of 25.2%. Cigarettes were more affordable (defined according to the cost of cigarettes relative to per capita income) in 2016 than in 2008 in two countries, Indonesia and Viet Nam, but less affordable in 2016 than in 2008 in the Philippines.

Second, countries are required to eliminate exposure to second-hand tobacco smoke in all indoor workplaces, public places and transport. Bhutan (which has a total ban on tobacco) had the highest compliance rate (score: 10 out of a maximum 10), followed by Thailand (score: 7/10), while Indonesia (score: 1/10) had yet to scale-up compliance to protect the health of non-smokers.

Third, countries are required to introduce plain or standardized packaging or large graphic health warnings on all tobacco packages. Thailand and Sri Lanka were the two best-performing countries, as text and pictorial health warnings covered 85% and 80% of the front and back areas of cigarettes package, respectively. Health warnings covered only 40% of package areas in Indonesia.

Fourth, countries are required to enact and enforce comprehensive bans on tobacco advertising, promotion and sponsorship. Bhutan had the highest level of compliance with a score of 10 out of 10 each for direct and indirect bans, followed by Viet Nam with a compliance score of 10/10 for a direct ban and 6/10 for an indirect ban. Indonesia had the lowest score (1/10) on eliminating exposure to second-hand tobacco smoke; the country had no bans on direct advertising or sponsorship; and low compliance (score 3/10) on banning free tobacco distribution, 

Fifth, countries are required to implement effective mass-media campaigns to educate the public about the harms of smoking and second-hand smoke. All countries except Bhutan and Sri Lanka had comprehensive campaigns in the media in 2014 and 2016.

### Alcohol control

There are three indicators in the *Global status report on alcohol and health 2018*, that were used to monitor progress on reduction of harmful use of alcohol.^19^

First, countries need to enact and enforce restrictions on the physical availability of retailed alcohol. The legal minimum age for on- and off-premise sales of alcoholic beverages in 2018 was the highest in Indonesia and Sri Lanka (21 years), followed by Bhutan, Philippines and Viet Nam (18 years), while Cambodia did not have a defined legal age. All countries in this study except Cambodia had introduced restrictions on the on- and off-premise sales of alcoholic beverages by timing or place, although these was not yet comprehensive.^19^

Second, countries need to enact and enforce bans or comprehensive restrictions on exposure to alcohol advertising in all types of media, product placement, sponsorship and sales promotion, and implement health warning labels on alcohol advertisements and containers. We found that almost all countries had introduced regulations on advertising for all categories of media except on alcohol drinks containers.

Third, countries need to increase excise taxes on alcoholic beverages including beer, wine and spirits. The *Global status report on alcohol and health 2018*^19^ does not provide detailed information, such as tax rates, trends of tax rates and changes of affordability of alcoholic beverages. However, most countries had imposed excise taxes for all alcoholic beverages, except on spirits in Bhutan. The available information would not be helpful for monitoring progress on changes of affordability and specific policy interventions.

### Unhealthy diet

The availability of a salt policy is currently the only indicator used by WHO to monitor progress on unhealthy diet.^21^ Salt policies cover four best buys interventions; (i) reformulating and setting target of salt in foods, (ii) promoting an enabling environment for lower sodium options, (iii) promoting behaviour change through media campaign, (iv) implementing front-of-pack labelling. Thailand had introduced a salt and sodium reduction policy for 2016–2025, focusing on labelling, legislation and product reformulation.^24^ In 2016, Thailand adopted national policies to reduce population salt and sodium consumption, in the form of a voluntary salt reduction in processed food and snacks. Manufacturers who comply with the salt reduction recommendation (including those on fat and sugar) receive a healthier choice logo by the food and drug administration of the health ministry. A regulation was introduced in 2016 in Thailand, for mandatory package labelling (of salt, fat, sugar, energy and other contents) through the guideline daily amount. Bhutan and Sri Lanka have drafted salt reduction strategies, although an explicit policy on salt reduction was not yet available. Average daily salt intake was 10.8 g (in 2010) and 8.0 g (in 2012) in Thailand and Sri Lanka, respectively,^26^ which is more than the 5 g recommended by the WHO.^27^ Population behaviour change actions, such as creating awareness on high salt intake and empowering people to change their behaviours, had been introduced in Bhutan and Sri Lanka.

### Physical activity

Implementing public education and awareness campaigns is the indicator for monitoring progress of promoting physical activity.^21^ By 2016, Cambodia and Viet Nam had not implemented any programme activities that support behavioural change in the previous 5 years. The *Global action plan on physical activity (2018–2030)*, adopted by World Health Assembly resolution WHA71.6^28^ in May 2018, urged the WHO Member States to implement the promotion of physical activity and requested the WHO to develop global monitoring and reporting systems.

### Health-systems response

Two indicators are proposed for monitoring health-systems response to noncommunicable diseases: availability of treatment guidelines and availability of essential medicines at primary level facilities.^21^ Access to essential medicines supports reduction of premature mortality in SDG target 3.4.

By 2016, all seven countries had developed evidence-based national guidelines for the management of four major conditions through a primary health-care approach, although there was no detail on the scope and contents of guidelines. Three countries, Philippines, Sri Lanka and Thailand, reported that more than 50% of their primary health-care facilities offered cardiovascular risk management of patients at risk of heart attack and stroke. The remaining four countries reported fewer than 25% of their primary care facilities offered these services.

Indonesia and Sri Lanka reported that 11 out of 12 priority noncommunicable diseases medicines were available in more than 50% of their primary care facilities. Viet Nam and Cambodia needed to scale-up availability of these medicines, as only 2/12 and 3/12 medicines for noncommunicable diseases were available, respectively.

In addition to the cross-country analysis in Table 2, Box 1 provides a synthesis of intra-country analysis of their noncommunicable diseases interventions, achievements and gaps.

Box 1 **Best-buy interventions for the prevention and control of noncommunicable diseases: summary of achievements and gaps in seven Asian countries in July 2018**

**Bhutan**

Although smoking is illegal in Bhutan, the current prevalence of tobacco use among young people and adults is estimated to be 30.2% and 7.4%, respectively in 2016. The country has good performance in ensuring smoke-free public spaces (compliance score 10/10) and total bans on tobacco advertising, promotion and sponsorship. Although excise taxes and restrictions on the availability and advertising of alcohol are in place, the legal minimum age for sales of alcohol beverage (18 years old) is the lowest among the seven countries. Bhutan is developing strategies on reduction of daily salt consumption and promotion of physical activity. While clinical guidelines for the management of four major noncommunicable diseases are produced, only four out of 12 essential medicines for management of these diseases are available in more than 50% of primary care facilities.

**Cambodia**

Tobacco control policies need considerable improvement. The tobacco tax rate is the lowest among the seven countries, 25.2% of the retail price. No price changes between 2008 and 2016 means that cigarettes are affordable by the WHO definition.^18^ There is room to strengthen compliance on smoke-free public spaces, increase the health warning areas on cigarette packages (55%) and introduce a ban on indirect marketing promotions. Cambodia needs to introduce a legal minimum age for sale of alcoholic beverages and to restrict alcohol availability, limit daily salt consumption and promote physical activity. The country needs to scale-up the availability of essential medicines in primary care facilities.

**Indonesia**

A very high prevalence of tobacco use was reported in Indonesia; 12.7% of young people and 64.9% of men are current tobacco users. Though not a State Party to the WHO Framework Convention on Tobacco Control, the government needs to increase the low tobacco tax rate (57.4%) and make cigarettes less affordable to discourage new smokers, scale-up the current low level (score 1/10) of compliance on smoke-free public spaces, increase health warning areas on cigarette packages (currently 40% of front and back areas), and introduce a ban on advertising and market promotion. Alcohol consumption is religiously prohibited and legal measures to reduce alcohol consumption are well-implemented. The legal minimum age for purchase is 21 years and restrictions of the times and places of alcohol availability and advertising are in place. Indonesia has yet to introduce a salt reduction policy. Health systems are responding well as 11 out of 12 essential medicines for noncommunicable diseases are available in primary care facilities.

**Philippines**

Although cigarettes were less affordable in 2016 than in 2008, the Philippines needs to further increase the tax rate (62.6%), improve compliance on smoke-free environments, increase the size of health warnings (50% of cigarette package areas) and increase compliance on bans on advertising and promotion. The country also needs to review the current legal minimum age (18 years) for sales of alcoholic beverages, introduce policies to limit daily salt consumption and increase the availability of essential medicines for clinical management in primary health care.

**Sri Lanka**

Although the tobacco tax rate is 62.1%, the lack of regular tax increases means that cigarettes are still affordable. Sri Lanka needs to further strengthen compliance on smoke-free environments and bans on advertising and promotion. The country is on the right path towards implementing salt reduction strategies and promotion of physical activity. Due to the strong emphasis on primary health care in the country, the availability of essential medicines at the primary care level has been ensured.

**Thailand**

Tobacco control is well-implemented with a high tax rate in place (73.5%), health warnings on 85% of the back and front package areas (which ranks third globally^1^) and comprehensive regulations on advertising, market promotion and sponsorship. However, Thailand needs to improve compliance on smoke-free environments. Due to Thailand’s policy of universal health coverage, nine essential medicines for noncommunicable diseases are available at primary care facilities.

**Viet Nam**

Lack of regular increase in tax has resulted in more affordable cigarettes in 2016 than in 2008. Viet Nam therefore needs to increase its tax rate (35.7%) improve compliance on smoke-free environments and increase health warnings from the current 50% of package areas. Increasing the current minimum legal age for sales of alcoholic beverage (18 years) may prevent youth drinking. The country needs to introduce policies to reduce daily salt intake (currently only dietary guidelines are available and there is no front-of-package labelling^1^), promote physical activity, and ensure more essential noncommunicable diseases medicines are available in primary care facilities.

Note: See Table 2 for more details and data sources. Affordability of cigarettes is defined by the percentage of per capita gross domestic product required to purchase 2000 cigarettes of the most sold brand.^18^

### Institutional capacity

Translating the UN General Assembly resolutions into interventions with good outcomes requires institutional capacity to deliver these political promises. We obtained information directly from health ministries on their institutional capacities for noncommunicable diseases (Table 3).

**Table 3:** Institutional capacity for the prevention and control of noncommunicable diseases in seven Asian countries in July 2018

Indicator	Bhutan	Cambodia	Indonesia	Philippines	Sri Lanka	Thailand	Viet Nam
No. of full-time equivalent technical professional staff in noncommunicable diseases unit under health ministry^a^	4	7	16	19	41	39	7
No. of full-time equivalent staff in health ministry for tobacco control^25^	14	6	12	3	10	41	20
National funding for noncommunicable diseases prevention, promotion, screening, treatment, surveillance, monitoring and evaluation, palliative care and research^a^	Yes	Yes, except research budget	Yes	Yes	Yes	Yes	Yes
Sources of funding for noncommunicable diseases and their risk factors^a^	Government budget and donors	Government budget, donors and social protection schemes	Government budget and health insurance	Government budget and health insurance	Government budget and donors	Government budget, health insurance and Thai Health Promotion Foundation	Government budget, health insurance, donors and earmarked tobacco tax
Government expenditure on tobacco control (year), US$^25^	23 000 (2014)	22 200 (2008)	882 414 (2008)	21 739 (2007)	462 235 (2016)	892 359 (2015)	12 000 000 (2016)

US$: United States dollar.

^a^ Personal communication with health ministries.

All seven countries had designated a unit or equivalent body in their health ministry with responsibility for noncommunicable diseases. The number of full-time equivalent professional staff in the unit ranged from four in Bhutan to 41 in Sri Lanka. As required by the WHO FCTC reporting, the number of full-time equivalent for tobacco control ranged from three in the Philippines to 41 in Thailand.

Funding for noncommunicable diseases interventions (including prevention, promotion, screening, treatment, surveillance, monitoring and evaluation, capacity-building, palliative care and research) were available in all seven countries, except for a research budget in Cambodia.

Data were not available on annual spending on noncommunicable diseases, although all countries relied on government budget allocation and a small proportion of donor funding. Health insurance subsidized the cost of treatment in Cambodia, Indonesia, Philippines, Thailand and Viet Nam. A 2% additional surcharge from a tobacco and alcohol excise tax was earmarked and managed by the Thai Health Promotion Foundation^29^ for comprehensive interventions for noncommunicable diseases and other risk factors. An earmarked tax from alcohol and tobacco sales in the Philippines is used to subsidize health care in general, for the 40% of the population who are low income, and Viet Nam has earmarked the tobacco tax for the tobacco control programme. A great variation on annual spending on tobacco control was noted in these countries, ranging from US$ 21 739 in the Philippines to US$ 12 million in Viet Nam (Table 3).

## Challenges

### Implementation gaps

Institutional capacity assessment in the seven countries is constrained by several limitations. Disaggregated information on the skill-mix of technical staff in countries’ health ministry noncommunicable diseases units, and staff turnover rate, are not routinely recorded and reported. This evidence is critical for analysing gaps and strengthening the capacity of noncommunicable disease units. In the countries we analysed, information was also lacking on government spending on health promotion interventions. Using the WHO Health Accounts database,^30^ we estimate that the global average investment on health promotion and public health interventions worldwide in 2012 was 4.3% of current per capita health spending (US$ 38.6 of US$ 989.2). Despite the well-established monitoring and evaluation system of the WHO FCTC, data on expenditure for tobacco control is not routinely updated for many countries. For example, the latest expenditure data on tobacco control in the Cambodia, Indonesia and Philippines were outdated, from 2008, 2008 and 2007, respectively.

Taxation on tobacco and alcohol has not reached the global targets in these seven countries, mainly due to the lack of multisectoral action to enforce legislative decisions on taxing these harmful products and counteracting industry interference. These concerns were highlighted by the UN Interagency Task Force on noncommunicable diseases conducted in these countries.^31^ Furthermore, primary prevention efforts in the seven countries are hampered by weak regulatory capacities, inadequate legal consequences for law violation and conflicts of interests among government officials. Regulatory gaps were illustrated by poor enforcement of smoke-free environments or of bans on tobacco advertising and promotion. Besides Sri Lanka and Thailand, integration of noncommunicable disease interventions at the primary care level need to be strengthened in the remaining five countries, to ensure essential medicines for clinical management, prevention of complications and premature mortality. Funding gaps for noncommunicable diseases, as reported by health ministries, remain an important national agenda in these countries and the governments need to invest more on effective interventions such as the recommended best buys, intersectoral actions and health-system responses for noncommunicable diseases.

Another possible explanation for insufficient progress of noncommunicable diseases prevention policy is industry interference.^32^ There is evidence from other countries that the tobacco,^33^^–^^35^ alcohol,^36^ food and beverage industries^37^ use tactics to interfere with policies aimed at reducing consumption of their unhealthy products. 

The South East Asia Tobacco Control Alliance has pioneered the Tobacco Industry Interference Index to monitor tobacco industry actions.^38^ Viet Nam and Indonesia have demonstrated high levels of industry interference,^39^ with marginal improvement between 2015 and 2016, which may be linked to the lack of progress on tobacco control in both countries. The tobacco industry has been more effective in promoting their products than governments have been in implementing effective interventions, as reflected by the slow progress in tobacco control efforts in the countries we analysed. In Indonesia, a non-State Party to the WHO FCTC, the level of tobacco industry interference is the highest, although the health ministry is drafting guidelines for interaction with the tobacco industry.^40^ Article 5.3 of the WHO FCTC guides State Parties to protect their tobacco control policies from the vested interests of the tobacco industry.^41^ Global experience shows how the tobacco industry’s corporate social responsibility activities are a platform for government officials to participate directly in the industry’s activities. All countries in this study have yet to establish procedures for disclosing interactions between governments and the industry.

Industry interference with government policies is further highlighted by Thailand’s experience in introducing an excise tax on beverages containing sugar in 2017,^42^ where the government faced resistance by the Thai Beverage Industry Association that challenged the links between obesity and drinking soda.^43^

To address the commercial determinants of noncommunicable diseases and policy interference by industries, countries require improved governance, political leadership and a whole-of-government approach to making legislative decisions on taxation and strengthening regulatory capacities.

### Monitoring and evaluation gaps

The existing systems for surveillance of health risks, including the prevalence of smoking, alcohol per capita consumption, daily salt intake and levels of physical inactivity, need strengthening, standardization and integration for comprehensive noncommunicable diseases policies to be formulated. Integrated household surveys such as the *STEPwise approach to surveillance*^44^ or equivalent should cover all noncommunicable diseases risks in one survey.

The lack of global standardized detail reporting on alcohol control hampers countries from monitoring and advancing the alcohol control agenda; for example, monitoring tax rates against the preferred level of tax rate, similar to the FCTC MPOWER report.^18^ Estimations of daily salt intake requires laboratory testing to quantify 24-hour urinary sodium excretion,^45^ and only a few countries worldwide conduct such surveys.^46^^,^^47^ The burdensome 24-hour collection of urine can be replaced by urine spot testing,^48^ which is more practical and less costly. Salt intake using spot urine samples can provide countries with a good indication of mean population salt intake.^49^ The level of daily salt intake is a powerful message for policy advocacy in educating the public and benchmarking with international peers. Monitoring measures for unhealthy diet reduction need to be more comprehensive. Such monitoring needs to cover people’s consumption of trans-fat and sugar-sweetened beverages; policy interventions such as introduction of sugar-sweetened beverages taxes and bans on trans-fat in food; and the food industries’ responses and adherence to policy.

Learning from the FCTC global tobacco epidemic report,^18^ the WHO and international partners should develop a standardized, comprehensive monitoring tool on alcohol, salt, unhealthy food, physical activity and primary health-care readiness to provide noncommunicable diseases services. The indicators in the country capacity survey^24^ are inadequate to drive health-systems responses to noncommunicable diseases.

## Conclusion

Our survey identified more challenges than achievements in these seven Asian countries, although some progress has been made since implementing their national action plans on noncommunicable diseases control. Key underlying barriers for insufficient progress of noncommunicable disease policy are the lack of institutional capacities of noncommunicable disease units in managing action across different sectors; inadequate investment on primary prevention; and inadequate health-systems responses on clinical management. The multifactorial nature of noncommunicable disease requires coordinated health action across sectors within and outside the health system, including tax policies, health policies, food policies, transport and urban design. To overcome implementation gaps, governments need to improve the coordination of noncommunicable diseases units with other sectors, invest more in effective interventions such as the WHO recommended best buys, and improve monitoring and evaluation capacities.
